# Supplementary material for: “People in my life just play different roles”: A retrospective qualitative study of friendships among young adults who self-harmed during adolescence
Source: PLoS One. 2025 Apr 25;20(4):e0320206. doi: 10.1371/journal.pone.0320206 (PMC12027263; doi:10.1371/journal.pone.0320206)
Supplement: S1 File — Summary of interview schedule. (PDF) [file pone.0320206.s001.pdf]

S1 File. Interview schedule

Summary interview schedule

1. **Initiation questions i.e., self-harm onset**
   First choose and sort cards based on your experience of one of your first episodes of self-harm. It's up to you to decide which cards to include and how to sort them, there are no right or wrong answers. The following questions are in regards to the first CaTS configuration.

   - How would you describe one of the first times you self-harmed?
   - How did you learn about/come across the idea of self-harm?
   - Which cards do you consider more important/salient to one of your first experiences of self-harm?
   - What were your friendships like before and after self-harming for the first time?
   - Can you tell me more about this card e.g., isolation from others?
     How important was this particular event/card e.g., I got involved with a new group of friends who self-harm?
   - Did you tell someone about self-harm? Who?
     If anyone, what happened after you told them? How did they respond?
   - What role, if any, did your friends/peers have in your experience?

2. **Maintenance questions i.e., most recent self-harm**
   Now look at the cards in front of you and add/remove/change cards to represent one of the most recent times that you have self-harmed. It's up to you to decide which cards to include and how to sort them, there are no right or wrong answers. The following questions are in regards to the second CaTS configuration.

   - What made you self-harm again after the first time?
   - Thinking about a recent episode in which you self-harmed, what would you say is different, if anything, compared to when you started self-harming?
     *Which cards have you changed/moved/added and why?
   - Have your reasons/motivations to self-harm changed at all since you first started?
     *Yes/No and why?
   - Thinking about your friends/ peers, has anything changed from the first time to your more recent self-harm?
   - Have you told someone else about self-harm? Who? How did they respond?
   - How would you describe your sources of support during your most recent self-harm?

3. **Advice questions**
   - For you, what do you think could be done to better support you or others in your situation?
   - If you had your younger self in front of you, what three things would you like them to know.
   - If you had your friends in front of you, what three things would you like them to know.
