# Supplementary material for: “People in my life just play different roles”: A retrospective qualitative study of friendships among young adults who self-harmed during adolescence
Source: PLoS One. 2025 Apr 25;20(4):e0320206. doi: 10.1371/journal.pone.0320206 (PMC12027263; doi:10.1371/journal.pone.0320206)
Supplement: Attachment — Submitted filename: Reply to Reviewers v2.docx [file pone.0320206.s003.docx]

PONE-D-24-36301

“People in my life just play different roles” A retrospective qualitative study of friendships among young adults who self-harmed during adolescence

PLOS ONE

Dear Dr. Bilello,

Thank you very much for submitting your manuscript to PLOS ONE. After careful consideration, we feel that it has merit but does not fully meet PLOS ONE’s publication criteria as it currently stands. Therefore, we invite you to submit a revised version of the manuscript that addresses the points raised during the review process.

While there is much to admire in your paper, in particular, having read your paper and the referees' comments, I would like you to address carefully the comments of both referees, who suggest that your manuscript needs to discuss better the following points:

1) Greater consistency and clarity when discussing the types of friends involved in the study. 
2) Inconsistency in the terminology used to describe the participants' developmental stages throughout the manuscript. 
3) Think carefully about potential limitations to your study.
4) Further reflection on how the researcher’s position as a student may have influenced their interactions with participants, who were also students.

Please submit your revised manuscript by Dec 13 2024 11:59PM. If you will need more time than this to complete your revisions, please reply to this message or contact the journal office at <a href="mailto:plosone@plos.org">plosone@plos.org. When you're ready to submit your revision, log on to https://www.editorialmanager.com/pone/ and select the 'Submissions Needing Revision' folder to locate your manuscript file.

Please include the following items when submitting your revised manuscript:

- A rebuttal letter that responds to each point raised by the academic editor and reviewer(s). You should upload this letter as a separate file labeled 'Response to Reviewers'.
- A marked-up copy of your manuscript that highlights changes made to the original version. You should upload this as a separate file labeled 'Revised Manuscript with Track Changes'.
- An unmarked version of your revised paper without tracked changes. You should upload this as a separate file labeled 'Manuscript'.

If you would like to make changes to your financial disclosure, please include your updated statement in your cover letter. Guidelines for resubmitting your figure files are available below the reviewer comments at the end of this letter.

If applicable, we recommend that you deposit your laboratory protocols in protocols.io to enhance the reproducibility of your results. Protocols.io assigns your protocol its own identifier (DOI) so that it can be cited independently in the future. For instructions see: <https://journals.plos.org/plosone/s/submission-guidelines#loc-laboratory-protocols>. Additionally, PLOS ONE offers an option for publishing peer-reviewed Lab Protocol articles, which describe protocols hosted on protocols.io. Read more information on sharing protocols at <https://plos.org/protocols?utm_medium=editorial-email&utm_source=authorletters&utm_campaign=protocols>.

We look forward very much to receiving your revised manuscript and thank you for submitting your research to PLOS ONE.

With kind regards,

Professor Sriya Iyer
Academic Editor
PLOS ONE

[Note: HTML markup is below. Please do not edit.]

Dear Professor Sriya,

Thank you very much for the opportunity to re-submit and for your constructive editorial comments and the helpful peer review. We are delighted to re-submit the revised manuscript entitled *“People in my life just play different roles”: A retrospective qualitative study of friendships among young adults who self-harmed during adolescence”* to *PLOS One.* This retrospective qualitative study aimed to understand the relationship between friendships and self-harm and their co-evolution over time among adolescents. We focus on understanding the meaning of both friendships and self-harm, their interdependent nature and mutual influence. The present study sheds light on the lived experience of friendships and self-harm in a sample of university students who self-harmed during adolescence.

The reviewers made a number of well-informed, constructive comments which we are pleased to address in the following sections. Both reviewers expressed concerns around **language/ terminology** (Editor #1 and #2; Reviewers #1, #2) to describe developmental age and type of friends in the study. These comments have been addressed throughout the manuscript. First, we have decided to clarify the terminology around the population of interest (and to clarify the age range) by clearly defining these populations in line with available definitions in the introduction (i.e., adolescents and young people; WHO definition). However, where appropriate we have adopted broader terminology to avoid confusion (e.g., individuals, people, participants). Equally, we have clarified in the results section the type of friend most discussed by participants (i.e., school-based same-age friendships). There were additional major concerns regarding **limitations** and **reflexivity** (Editor #3 and #4; Reviewer #1)**.** The limitations section has been expanded to consider some additional concerns, including the sample and the representation of experiences (including a discussion around the concept of saturation). In turn, we have included a new section in the discussion (section 4.5) to include a more detailed discussion around reflexivity and how researchers’ characteristics influence the research process and interpretation of results.

Some minor comments have also been addressed in their corresponding section and are explained under each reviewers’ comments/ suggestions.

We hope that our manuscript is now suitable for publication PLOS One, but of course are willing to make further amendments should they be required, and look forward to hearing from you.

Yours sincerely,

D Bilello, E Townsend, M R Broome, S Burnett Heyes

Editor’s Comments:

1) Greater consistency and clarity when discussing the types of friends involved in the study.

Thank you for this suggestion. We accept this criticism regarding lack of clarity around the types of friends discussed in the study. The updated manuscript now clarifies that across most interviews, these were same-aged school-based friendships. We also note that where findings reference other types of friendships this will be specified.

REVISED MANUSCRIPT (p. 12, 264-266): “Participants primarily discussed in-person same-aged school-based friendships, with few mentions of online or older friends. Therefore, the following results mainly reference the former group unless otherwise specified.”

This particular point is addressed in more detail in response to a comment from Reviewer 1 comment (page 7-8).

2) Inconsistency in the terminology used to describe the participants' developmental stages throughout the manuscript.

We agree and have taken the reviewers’ comments into consideration when addressing this point to be clearer and more consistent in our terminology. To facilitate understanding and clarity regarding the terminology, we carefully define the specific age range we are considering in the introduction (i.e., adolescents and young people), consistent with established definitions (WHO). We have revised the manuscript to make sure the use of these words is appropriate (e.g., if studies only include adolescent samples we use the word adolescent; results which mainly discuss adolescent experiences use only the term adolescent). Also, to avoid confusion we have adopted broader terminology throughout the manuscript where appropriate (e.g., changing “adolescents”/“young people” to “participants”/“individuals”):

REVISED MANUSCRIPT (p. 3, lines 36-38): “According to the World Health Organisation, adolescence encompasses the period from 10-19 years old, while the term young people refers to individuals between 10-24 years old (WHO, 2001).”

3) Think carefully about potential limitations to your study.

In line with the reviewer’s insightful comments, we have expanded the limitations section to consider potential limitations of the sample and implications for interpreting findings.

REVISED MANUSCRIPT (p., lines 686-701): “The present findings have certain limitations. First, despite the temporal view we adopted, we explored only a limited period, from early/mid-adolescence to young adulthood. Recent research highlights the potential for earlier self-harm onset (i.e. during childhood) and maintenance beyond young adulthood (Geoffroy et al., 2022). Nonetheless, our adolescent/young adult focus provides in-depth insight on a period of key relevance, given typical ages of self-harm onset, peak and decline (Whitlock & Selekman, 2014).

Second, the size and characteristics of our sample may have implications for the findings presented. The sample size was determined based on the overall research aim of providing an in-depth exploration of individuals’ experiences and sense-making. For this aim and when using an RTA approach, smaller samples of 10 to 20 participants are typically recommended (Braun & Clarke, 2019b). Despite the richness of the data captured in this study, it is important to reflect on the limitations of our purposeful sampling approach in sufficiently capturing participants’ experiences. Specifically, the sample consisted of undergraduate psychology students who may be a relatively homogeneous group with similar demographic and sociocultural characteristics and whose unique cultural and institutional context may have introduced potential population bias. As such and as mentioned in prior sections, the self-harm experiences of this sample may have certain similarities in trajectory and processes of sense-making. Therefore, it is plausible that certain self-harm experiences – where participants displayed balanced evaluations of their current situation in relation to both self-harm and friendships – were over-represented in our findings. Potentially, individuals with more difficult or unresolved experiences in the past and present chose not to participate, with implications for interpreting the current findings.”

4) Further reflection on how the researcher’s position as a student may have influenced their interactions with participants, who were also students.

In response to this important point, we have added a new section in the discussion section to provide a detailed reflexive statement. In this section, we reflect upon the primary researcher’s characteristics (e.g., age, education background) and how this may have impacted the research process, the overall findings and their interpretation.

REVISED MANUSCRIPT (p. 28-29, lines 613-646):

**“4.5 Reflexivity and researcher’s position**

The use of qualitative methods inherently assumes that research is contextual and subjective (Dogson, 2019; Olmos-Vega, Stalmeijer, Varpio & Kahlke, 2023). This subjectivity and the researchers’ own positions, characteristics, experiences and their roles in shaping the research, should reflexively be considered (Macbeth, 2001; Dogson, 2019).

Particularly, my position as a doctoral student shaped the research in various ways across the different stages of the research process. First, being a student implied initial limited knowledge around the topic and how to best approach it. This informed the decision to allow participants to self-identify and describe their own self-harm and friendship experiences, without a priori constraints. This approach may have facilitated interviews by allowing participants to feel in control of the interview process and may have helped validate their experiences. Equally, this may have made the interview process more challenging in some cases due to the broad and non-restrictive nature of the questions. However, the use of the CaTS scaffolded the process for participants.

Additionally, similarities between myself and participants also shaped the research process and interpretation of findings. On one hand, being relatively similar in age and gender to most participants appeared to facilitate certain conversations and reduce power dynamics. In turn, it promoted relatability and shared understanding of the context in which certain self-harm experiences occurred (e.g., Tumblr, online friendships). On the other hand, similarities in age and educational background (i.e., psychology) may have shaped conversations in a particular way. During interviews, some participants appeared to use more clinical language and reasoning (e.g., conversations about warning signs/ symptoms of certain mental health disorders and how they manifested in themselves or others). Whilst this helped create mutual understanding, it also appeared to lead to more neutral and objective descriptions and sense-making, potentially detracting from more experiential aspects of participants’ accounts of their subjective experiences.”

Reviewers' comments:

Reviewer's Responses to Questions

**Comments to the Author**

1. Is the manuscript technically sound, and do the data support the conclusions?

The manuscript must describe a technically sound piece of scientific research with data that supports the conclusions. Experiments must have been conducted rigorously, with appropriate controls, replication, and sample sizes. The conclusions must be drawn appropriately based on the data presented.

Reviewer #1: Yes

Reviewer #2: Yes

2. Has the statistical analysis been performed appropriately and rigorously?

Reviewer #1: N/A

Reviewer #2: N/A

3. Have the authors made all data underlying the findings in their manuscript fully available?

The [PLOS Data policy](http://www.plosone.org/static/policies.action#sharing) requires authors to make all data underlying the findings described in their manuscript fully available without restriction, with rare exception (please refer to the Data Availability Statement in the manuscript PDF file). The data should be provided as part of the manuscript or its supporting information, or deposited to a public repository. For example, in addition to summary statistics, the data points behind means, medians and variance measures should be available. If there are restrictions on publicly sharing data—e.g. participant privacy or use of data from a third party—those must be specified.

Reviewer #1: No

Reviewer #2: Yes

4. Is the manuscript presented in an intelligible fashion and written in standard English?

PLOS ONE does not copyedit accepted manuscripts, so the language in submitted articles must be clear, correct, and unambiguous. Any typographical or grammatical errors should be corrected at revision, so please note any specific errors here.

Reviewer #1: Yes

Reviewer #2: Yes

5. Review Comments to the Author

Please use the space provided to explain your answers to the questions above. You may also include additional comments for the author, including concerns about dual publication, research ethics, or publication ethics. (Please upload your review as an attachment if it exceeds 20,000 characters)

**Reviewer #1**: OVERALL SUMMARY

Thank you for the opportunity to peer-review this important manuscript on the impact of friendships among young adults who self-harmed during adolescence. The paper addresses an underexplored topic in self-harm research, offering valuable insights into the interrelationship between friendships and self-harm, and the complexities involved.

The study’s findings emphasise that friendships not only influence self-harm behaviours but are also shaped by them, which is an important contribution to the field. The use of semi-structured interviews within a retrospective qualitative design, alongside a modified CaTS sort task and reflexive thematic analysis, constitutes a strong and appropriate methodology for capturing the subjective experiences of participants. It was particularly commendable to see the focus on both the onset and maintenance of self-harm in relation to friendships. The CaTS-AF task was used effectively to capture these temporal aspects, and the researchers made full use of this tool to enhance their analysis. Overall, the study identifies relevant and significant themes, and the interpretation of the findings is well-grounded, offering fresh perspectives on the connection between adolescent friendships and self-harm.

Thank you very much for your carefully considered, insightful and positive comments on our paper. We really appreciate your detailed constructive feedback on the research and its importance. Your suggestions have been extremely valuable in concisely addressing various issues, resulting in a clearer more robust report. Below we highlight the various changes made to address your concerns.

MAJOR CONCERNS

However, I have a few concerns that might be considered more “major”.

1) There needs to be greater consistency and clarity when discussing the types of friends involved in the study. It’s not always clear whether the focus is on same-age friends, online friends, or offline friends, as these distinctions are not consistently made in the methods or results. For example, the method section on line 136 notes that the interview schedule included questions about participants’ “offline and online friend relationships,” but in the results, there isn’t always clarification about which type of friend is being referred to, assuming participants specified this at all. At times, same-age peers are mentioned, but it’s not explicitly outlined whether this is the primary type of friend captured throughout the study. If the intention is to refer to "friends in general" unless otherwise specified, this should be made clear in the results, particularly since the majority of interviewees appear to discuss same-age friends. For example, in the discussion, the phrase "given their status as same-aged peers" (line 529) is used, but it’s unclear if participants consistently referred to same-aged peers throughout the study. This ambiguity could be addressed by more clearly specifying in the methods and results which types of friends—whether peers, online friends, offline friends, or same-age friends—are being discussed. If there were different types, it might be important to reflect on this in the discussion.

Thank you for the opportunity to clarify.

In the study, we allowed participants to self-identify for both friendships and self-harm, and we did not ask them to define or categorise their friendships (to avoid imposing restrictive definitions). As a result, the interviewer did not ask participants to define their friend context nor their friend characteristics (e.g., age, online vs offline) at any point throughout the interview. These were only known if participants explicitly mentioned such characteristics. Nonetheless, based on contextual information provided throughout each interview (i.e., indirect evidence), most interviews reference same-aged offline friends (most were from school and face to face, with some also being online and outside of school).

To address this, we have removed restrictive statements about types of friends discussed and we have clarified that most friendships are same-aged, school-based friendships.

REVISED. MANUSCRIPT (p.8, line 166): “~~offline/online~~ friend relationships and the role of friends”

REVISED MANUSCRIPT (p. 12, lines 264-266): “Participants primarily discussed offline same-aged school-based friendships, with few mentions of online or older friends. Therefore, the following results mainly reference the former group unless otherwise specified.”

2) There is some inconsistency in the terminology used to describe the participants' developmental stages throughout the manuscript. Terms such as "adolescent" "young adult" "young person” are used. Given that adolescence is now often considered to extend up to age 24 (e.g., Sawyer et al. 2018 “the age of adolescence”), university students can still technically fall within this period. And given that the mean age of participants was 19 (with a range of 18 – 20) it might help to clarify that the developmental stage being discussed is still adolescence, but with self-harm recollected from early adolescence. This would ensure alignment with the current understanding of adolescence as extending into the mid-20s.

Thank you for the observation and we agree regarding inconsistencies. The different terminology as you rightly pointed out was mainly to differentiate between participants’ past experiences (in adolescence) and their present reflections (young adulthood). To ensure clarity regarding the terminology and the time period being discussed, we provide definitions of the specific age range considered in the introduction based on WHO definitions of adolescents and young people.

These terms are now used consistently throughout the updated manuscript as appropriate (e.g., if studies only include adolescent samples, we use the word adolescent; since our results mainly focus on adolescent experiences, we use the term adolescent in the results section). However, throughout the rest of the manuscript we have adopted more neutral terminology where possible (e.g., changing “adolescent” or “young people” for “participant” or “individual”):

REVISED MANUSCRIPT (p. 3, lines 36-38): “According to the World Health Organisation, adolescence encompasses the period from 10-19 years old, while the term young people refers to individuals between 10-24 years old (WHO, 2001).”

3) The limitations section could be expanded to address some important aspects that are currently missing. At present, the section is brief, only spanning two paragraphs, but there are several additional limitations that could be considered. For instance, the fact that the sample consists entirely of university students introduces an undergraduate population bias. The unique cultural and institutional context of the university may shape the participants' experiences and responses, and this should be acknowledged. While qualitative research is not focused on large sample sizes, but rather on the concept of data "saturation," it could still be valuable to address this explicitly in the limitations section. A brief comment on how saturation was approached or achieved would strengthen the methodological transparency of the study.

Thank you for your comment. We have now expanded the limitations section in order to include a more detailed reflection of limitations within the study. Based on your suggestions, we consider the relatively homogeneous sample in our study and how this may have impacted the conclusions drawn from the study.

As for data saturation, the approach we have utilised in the present study, namely Reflexive Thematic Analysis (Braun & Clarke, 2019), does not necessarily advocate for the use of data saturation. Instead, it assumes that themes and meanings are typically developed from the data collected and the process of interpretation (Braun & Clarke, 2021). Therefore, pre-establishing sample size is a difficult task when adopting this approach. For this, we considered typical recommendations for studies using RTA, participant availability/ participation, richness of the data collected, etc. This has been briefly reflected upon alongside potential limitations of our approach within the limitations section:

REVISED MANUSCRIPT (p., lines 686-701): “The present findings have certain limitations. First, despite the temporal view we adopted, we explored only a limited period, from early/mid-adolescence to young adulthood. Recent research highlights the potential for earlier self-harm onset (i.e. during childhood) and maintenance beyond young adulthood (Geoffroy et al., 2022). Nonetheless, our adolescent/young adult focus provides in-depth insight on a period of key relevance, given typical ages of self-harm onset, peak and decline (Whitlock & Selekman, 2014).

Second, the size and characteristics of our sample may have implications for the findings presented. The sample size was determined based on the overall research aim of providing an in-depth exploration of individuals experiences and sense-making. For this aim and when using an RTA approach, smaller samples of 10 to 20 participants are typically recommended (Braun & Clarke, 2019b). Despite the richness of the data captured in this study, it is important to reflect on the limitations of our purposeful sampling approach in sufficiently capturing participants’ experiences. Specifically, the sample consisted of undergraduate psychology students who may be a relatively homogeneous group with similar demographic and sociocultural characteristics and whose unique cultural and institutional context may have introduced potential population bias. As such and as mentioned in prior sections, the self-harm experiences of this sample may have certain similarities in trajectory and processes of sense-making. Therefore, it is plausible that certain self-harm experiences – where participants displayed balanced evaluations of their current situation in relation to both self-harm and friendships – were over-represented in our findings. Potentially, individuals with more difficult or unresolved experiences in the past and present chose not to participate, with implications for interpreting the current findings.”

4) Lastly, it would be useful to include further reflection on how the researcher’s position as a student may have influenced their interactions with participants, who were also students. This shared status could have affected the rapport between the researcher and participants, as well as the dynamics of the interviews. For instance, participants might have felt more comfortable or, conversely, may have withheld certain views based on perceived similarities or differences. On line 200, it is noted that a reflective diary was kept to account for the researcher’s impact during interviews and the analysis process, which is an important practice. However, it would be valuable to expand on this reflection in the discussion section. Even a brief mention of the specific insights or challenges arising from the researcher’s reflexive process could provide important context for interpreting the findings. If word count is a concern, this discussion could be summarised in one or two sentences within the main text, with more detailed reflections placed in the supplementary materials. A comment on reflexivity in the discussion, whether brief or expanded, would strengthen the study’s rigor by demonstrating an awareness of how the researcher’s positionality may have shaped the research process.

This is an excellent point. We have added a new section in the discussion based on your suggestion to capture our reflections around researcher’s positionality and its impact in the data collection/ interpretation process.

REVISED MANUSCRIPT (p. 28-29, lines 613-646):

**“4.5 Reflexivity and researcher’s position**

The use of qualitative methods inherently assumes that research is contextual and subjective (Dogson, 2019; Olmos-Vega, Stalmeijer, Varpio & Kahlke, 2023). This subjectivity and the researchers’ own positions, characteristics, experiences and their roles in shaping the research, should reflexively be considered (Macbeth, 2001; Dogson, 2019).

Particularly, my position as a doctoral student shaped the research in various ways across the different stages of the research process. First, being a student implied initial limited knowledge around the topic and how to best approach it. This informed the decision to allow participants to self-identify and describe their own self-harm and friendship experiences, without a priori constraints. This approach may have facilitated interviews by allowing participants to feel in control of the interview process and may have helped validate their experiences. Equally, this may have made the interview process more challenging in some cases due to the broad and non-restrictive nature of the questions. However, the use of the CaTS scaffolded the process for participants.

Additionally, similarities between myself and participants also shaped the research process and interpretation of findings. On one hand, being relatively similar in age and gender to most participants appeared to facilitate certain conversations and reduce power dynamics. In turn, it promoted relatability and shared understanding of the context in which certain self-harm experiences occurred (e.g., Tumblr, online friendships). On the other hand, similarities in age and educational background (i.e., psychology) may have shaped conversations in a particular way. During interviews, some participants appeared to use more clinical language and reasoning (e.g., conversations about warning signs/ symptoms of certain mental health disorders and how they manifested in themselves or others). Whilst this helped create mutual understanding, it also appeared to lead to more neutral and objective descriptions and sense-making, potentially detracting from more experiential aspects of participants’ accounts of their subjective experiences.”

MINOR SUGGESTIONS

Additionally, below are some minor suggestions specific to different sections of the manuscript, that may help to improve it further.

TITLE/ABSTRACT
• Title could better reflect the study by saying “role of friendships” e.g., [“People in my life just play different roles” A retrospective qualitative study of the role of friendships among young adults who self-harmed during adolescence”]

Thank you for your suggestion. We believe maintaining the original title ““People in my life just play different roles”: A retrospective qualitative study of friendships among young adults who self-harmed during adolescence” may be more suited in capturing the variety of themes in this study.

Whilst different friend “roles” or different friendships are discussed (as captured in the quote used for the title), the themes more broadly consider various aspects of friendships not limited to friends’ roles only (e.g., friendship characteristics, how SH influenced friendships).

• Abstract – lines 9/10 – might be better to have the (M=19.09; SD= 0.70; M=2, F=9) after the phrase young adults – because I read that as though the age they self-harmed was a mean age of 19.09 rather than that being the age of the sample.

Completely agree with this observation, thank you for noticing. This has now been corrected.

REVISED MANUSCRIPT (p.2, lines 9-10): “to explore the experiences of 11 young adults (M=19.09; SD=0.70; M=2, F=9) who self-harmed during adolescence.”

• Line 15 , could say “but they acquire” rather than “these acquire”.

This has now been corrected.

REVISED MANUSCRIPT (p.2, lines 15-16): “Furthermore, not only are friendships shaped by self-harm, but they acquire specific meanings…”

INTRODUCTION
• Line 56– maybe add in what friendships are defined as in the first place e.g., same age friends? Perhaps just outlining exactly what is meant by friendship in this context (this links in with the more ‘major’ concern comment 1 above).

Following your comment we have added a very broad definition of friendships and their roles during adolescence (in line 56). Additionally, we also explicitly clarify that friendships in this study mainly refer to in-person same-aged school-based friendships.

REVISED Manuscript (p. 4, line. 56-64): “Friendships are typically considered positive, voluntary and reciprocal informal relationships (Wrzus et al., 2015). However, these evolve over time and follow different trajectories as well as acquiring distinct roles and characteristics across the lifespan, in line with individuals’ developmental needs (Ajrouch et al., 2023; Pica & Verno, 2012). During adolescence, friendships gain importance as primary sources of companionship, emotional and social support as well as key influences on development and behaviour (Poulin & Chan, 2010; Dryuburgh et al., 2021; Bagwell & Schmidt, 2013).”

REVISED MANUSCRIPT (p. 12, lines 264-266): “Participants primarily discussed offline same-aged school-based friendships, with few mentions of online or older friends. Therefore, the following results mainly reference the former group unless otherwise specified.”

• On line 97, the research aim states “Describe participants’ perceptions of the role, importance and impact of friends on experiences of self-harm over time”. However, the study is also focused on exploring what the “meaning” of friendship in relation to self-harm is too, so that could be added/reflected in the aims. Also a suggestion - wonder if the aim stating “explore how friendships… were shaped by self-harm” the phrasing might suggest an assumption that self-harm shaped friendships, rather than an open exploration of all possible dynamics between self-harm and friendships i.e., implies that you are already expecting to find this. I appreciate that data has already been collected and analysed and this may have been your aim, but if your aim was intended to be more neutral and it is not too late to refine the wording for clarity and neutrality - instead of “how friendships were shaped by self-harm” - a more neutral phrasing could be “explore the relationship between self-harm and friendships” or “explore how self-harm and friendships interact”, which doesn’t assume a one-way effect.

This is a great point. We do focus on meaning throughout the study, therefore this needs to be (and has been) added to the aims.

REVISED MANUSCRIPT (p. 6, lines 104-105): “a) Describe participants’ perceptions of the meaning, role, importance and impact of friends on experiences of self-harm over time”

In terms of your second point, in the study we adopted a more open/exploratory aim consistent with the current literature (Bilello et al., 2024; Hall & Melia, 2023). Hence, we were open to friends influencing self-harm, self-harm influencing friendships and/or finding no influence between the two. To reflect this more appropriately and following your advice, we have adopted a more neutral wording for the second aim:

REVISED INTRODUCTION (p.6, line 106): “b) Explore how friendships and self-harm evolved and co-evolved over time.”

METHODS

• Line 128 – 133 in the study materials section “Depending on their preference, participants were given access to the CaTS-AF through a link to an online visual collaborative space (Mural), or received a paper copy by post. Participants were instructed to familiarise themselves with the CaTS-AF, pick cards that were relevant to their experiences (onset and most recent self-harm) and, if needed, write their own cards for experiences that were not already represented. Participants placed the selected cards along the timeline, and used stickers to identify cards they did not wish to discuss (Figure 2)” might be better placed in the procedure section on/after line 153.

Agree with this suggestion, we have now moved this information into the procedure section.

REVISED MANUSCRIPT (p.8, lines 156-164): “…participants completed a consent form and the baseline demographic questionnaire on Qualtrics and were sent the CaTS-AF before the next session. Depending on their preference, participants were given access to the CaTS-AF through a link to an online visual collaborative space (Mural), or received a paper copy by post. Consenting participants were then invited to a second and final Zoom session, in which the interview took place. Participants were instructed to familiarise themselves with the CaTS-AF, pick cards that were relevant to their experiences (onset and most recent self-harm) and, if needed, write their own cards for experiences that were not already represented. Participants placed the selected cards along the timeline, and used stickers to identify cards they did not wish to discuss (Figure 2).”

• Line 89 “here we adapted this tool and framework to the study of friendship and self-harm to consider the dynamics of friendships and self-harm from a first-person perspective” – not quite clear exactly how you adapted it – what was the process of adapting it? You mention on line 124 “adding 18 new social cards based on team discussions and relevant literature” but could maybe add a bit more about how these cards were derived/any things that were discussed but maybe did not go in there and if there was any kind of consensus approach to this.

This section has been expanded based on your suggestion to provide additional information about the process of creating the cards alongside some examples.

REVISED MANUSCRIPT (p.7, lines 138-149): “The process consisted of an initial literature search of relevant studies considering social and friendship dimensions of self-harm (Edmondson et al., 2016; Heath et al., 2009). [*author initials]* compiled an initial list of possible cards to be added which were compared against the original task. Most additions were based on varying the wording of original cards (e.g. to capture interpersonal factors, or to simplify or combine similar cards) while in some cases, cards were created following an iterative process whereby proposed statements were discussed, selected and refined through consensus within the research team. Wording of both new and modified cards was selected based on relevance to social dimensions noted in prior work. Examples include cards referencing social motivations and functions of self-harm (e.g., *I was trying to push people away, I got involved with a new group of friends who self-harmed, Someone was dismissive*). This resulted in 18 new ‘social’ cards and 19 modified versions of original cards, resulting in the final 116-card Card-sort Task About Friends (CaTS-AF). ”

• Line 169 “participants who responded emphasised positive aspects of participation (Dazzi et al., 2014)” – could state what they were/unpack this a bit more as it’s not quite clear what you mean. Also not sure if this citation should be here i.e., the reference is titled “Does asking about suicide and related behaviours induce suicidal ideation? What is the evidence?” - which may/or may not be relevant to this comment.

REVISED MANUSCRIPT (p. 9, lines 189-195): “Participants were contacted again after two weeks to share thoughts or comments regarding their experiences of the study. Participants who responded to the email suggested not being negatively affected by taking part, but rather they felt that their participation allowed them to reflect on their experiences. This is in line with prior findings that asking about self-harm in research studies does not necessarily impact participants negatively and indeed may have relatively positive and/or perceived therapeutic benefits (Dazzi et al., 2014). Follow-up comments were not included in data analysis.”

• Ethical considerations – The manuscript could benefit from more detail regarding how potential participant distress was handled, especially given the sensitive topic of self-harm. For example, it would be useful to clarify how the study followed up on participants who might have experienced distress during or after the interviews (e.g., line 145 mentions addressing potential distress). It would be helpful to specify what steps were taken to mitigate this, such as offering support or referrals, and whether any participants required follow-up. If relevant, including this in the results section would provide transparency about how such situations were managed

More information on procedures to ensure ethical standards have been added. It is also worth noting that the initial zoom session provided the researcher with an opportunity to give in-depth, concrete explanations of the study, including the types of questions that would be asked, and to allow participants to ask questions. This was an important part of our ethical considerations as it allowed participants to self-select out of the study before giving consent. In effect, following this session, some individuals decided not to participate possibly given they thought the contents of the interview may be distressing (see recruitment figure). This was an initial step to minimise distress.

MANUSCRIPT (p. 7-8, lines 176-179): *“*Participants took part in an initial 1:1 Zoom session with the primary researcher to discuss the aims and conduct of the study and to familiarise themselves with the sensitive content. If comfortable and eligible to take part”

REVISED MANUSCRIPT (p. 9, lines 178-189): “In order to maintain ethical standards in an online setting, several steps were taken. First, *[author initials]* checked-in with participants at various stages of the interview to ask them whether they felt comfortable to continue with the interview, whether they wanted to take a break or if they wanted to stop the interview altogether. This was typically done following CaTS completion and before/after specific questions. No participant chose to take a break or stop the interview, rather all stated they wished to continue.

Second, after the interview, all participants were debriefed and signposted to sources of support (e.g., university counselling services, helplines) and *[author initials]* engaged in informal conversation prior to closing the Zoom session to ensure participants were able to de-escalate from the potentially emotionally difficult conversation. Overall, no major distress was noted during the interview. Finally, participants were contacted again after two weeks to share thoughts or comments regarding their experiences of the study.”

• It would be beneficial to provide examples of the specific questions or prompts used in the interviews, to give readers a clearer understanding of how data was collected. Additionally, please clarify how the interviews were handled after data collection, particularly in terms of data storage. For instance, were the recordings deleted after transcription, and if so, what were the procedures to ensure data security? It would also be helpful to mention whether the interview guide evolved during the study and, if so, how changes were made throughout the course of data collection.

We have included some examples of questions that were asked during the interview in the Supplementary Information. We have also provided more information regarding the procedures to ensure data security.

Most changes during data collection were primarily in terms of selecting the prompts that worked best and refining questions based on specific cards that were consistently chosen by participants (e.g., isolation form others). Thus, the interview schedule did not undergo major changes, but rather there were slight refinements to the questions.

REVISED MANUSCRIPT (p.7, lines 149-163): “Semi-Structured interviews. The interview schedule included questions centred around participants’ self-harm experiences and trajectory, including: a) own understanding and experience of self-harm, b) friend relationships and the role of friends, c) characteristics and evolution of the friendship, and d) advice for supporting young people who self-harm (see supplementary information for interview schedule).”

As for more information regarding data storage, we have added this important information in the methods section.

REVISED MANUSCRIPT (P.9, 192-197): “Both arrangements were used as prompts in the semi-structured interview which was audio-recorded using encrypted electronic and physical audio-recording devices. Audio-recordings were transferred to a secure University server, while interview transcripts were stored in a locked cabinet in the University (for paper copies) or in an encrypted file in a password-protected folder on a secure laptop (for electronic copies). Recordings were deleted when transcription was complete.”

RESULTS

• Line 337: I may be misunderstanding the concept, but I’m unclear about how the "notion of isolation appears as a paradox given that participants also reported feeling alone and rejected by others." Feeling alone and rejected seems to support, rather than contradict, the idea of isolation. Could you clarify how isolation contrasts with other experiences, such as being physically present with others but still feeling emotionally isolated? This distinction could highlight how individuals can be around people yet still experience a sense of disconnection. If this is not the intended meaning, it might be worth reconsidering whether the term "paradox" is the most appropriate choice in the original sentence. If isolation leads to feelings of loneliness and rejection, then it doesn’t necessarily present a paradox. Alternatively, is the paradox meant to convey that adolescents feel isolated when, in fact, they are not—perhaps due to their friends perceiving them as the ones pushing others away? This could suggest a distorted perception of isolation. In the discussion on line 516, you mention that "first, feelings of shame, burdensomeness and thwarted belongingness are characteristically experienced in the context of self-harm” and interestingly, they also contribute to social withdrawal and isolation. This implies a paradox where young people who self-harm experience thwarted belongingness yet also choose to withdraw socially. If this is the case, it would be beneficial to unpack this idea more clearly in the results section to clarify what you mean by "paradoxical."

This is a very interesting point. As you suggest the paradox comes into play given that adolescents socially withdrew from others despite initially feeling thwarted belongingness or that no one cared. But as you rightly mention, these feelings and feelings of loneliness are further emphasised as a result of the act of isolating themselves from others which is an expected outcome (and not a paradox in this case). We think retaining the title gives nuance to these findings, yet we have unpacked the results to reflect this paradox more clearly and the consequences it may have for self-harm.

REVISED MANUSCRIPT (p. 18, lines 408-417): “Paradoxically, most participants suggested feeling lack of connection to others or that others did not care as the primary motivation for isolating themselves and pushing others away. However, this consequently amplified unwanted feelings of loneliness: “It’s not like I wanted to be on my own, I just thought I was” (P11):

“Some of my friends noticed (isolation), but I didn’t really take it seriously that they noticed. I think because I kind of managed to convince myself that nobody, like genuinely cared” (P21)

Overall, negative feelings appeared to contribute to isolation which further reinforced adolescents’ beliefs and distorted perceptions of themselves and others. Ultimately, this served to amplify their negative feelings in a cyclical manner. This highlights an important pathway towards self-harm maintenance and difficulties accessing support.

DISCUSSION
• See major concerns point 3 and 4.

These have been addressed in their corresponding section.

FIGURES
• Flow diagram – where it says "n=30" I initially interpreted that as you had posted 30 recruitment adverts. It could be clearer that it means 30 people responded to the advert? E.g., maybe put “Participants that responded to the recruitment advert posted in the research participant scheme, n =30” . Also not clear why some bits are capitilised e.g., Informed consent.

Thank you for your suggestion. We have changes this to reflect participants responding to the advert rather than the number of adverts posted. This also is in line with the wording in the other boxes. We have also revised the formatting of words to be consistent. See figure below.

• Figure 3 – should “self-harm” be a box around the circles to show self-harm as the context? Just a thought. Do ignore though if this isn’t what you are trying to capture here.

Thank you for your suggestion, we have now adapted the figure following your suggestion.

REVISED FIGURE

SMALL COMMENT ON GRAMMAR
• I apologise if this comes across as pernickety, but there are some phrases in the manuscript that could be refined for clarity and consistency. For example, in line 7 of the abstract, you state, 'the present study conducted…' It might be clearer to say, 'in the present study, we conducted…' because attributing the conducting to the people rather than the study enhances clarity. If you do change this, be consistent throughout the manuscript.

We have thoroughly checked the manuscript and identified some additional examples using this construction. We have now corrected these sentences. See some examples below:

REVISED MANUSCRIPT (p.2, line 7): “In the present study we conducted semi-structured..”

REVISED MANUSCRIPT (p.5, line 97): “In the present study we adopted a retrospective…”

REVISED MANUSCRIPT (p.5, line 102): “We adopted ~~The present study adopted~~ a retrospective temporal approach to explore experiences of friendships and self-harm during adolescence”

REVISED MANUSCRIPT (p.11, line 225): “We analysed the data ~~were analysed~~ using Reflexive Thematic Analysis”

REVISED MANUSCRIPT (p. 11 lines 235-236): “At this stage, we developed and applied interpretative codes ~~were developed and applied~~ to the transcripts, these informing initial themes.”

REVISED MANUSCRIPT (p.23; line 498-499): “In the present retrospective qualitative study we explored experiences of friendships and self-harm”

REVISED MANUSCRIPT (p. 25, line 538): “The qualitative temporal approach we adopted”

REVISED MANUSCRIPT (p. 26, line 581): “Finally, findings from the present study suggest~~s~~ that friendships acquire specific meaning”

REVISED MANUSCRIPT (p. 30, line 678): “In the present retrospective qualitative study we explored the experiences of friendships”

I hope you find this feedback helpful, and I wish the authors continued success in their future work!

**Reviewer #2**: Thank you for the opportunity to review your manuscript. It was very clear and well-written and I believe the topic is relevant and important. You did a nice of describing the study, clearly articulating the findings, and connecting your findings to current literature. I have just a couple of minor suggestions that may help with clarity and consistency. In some places you use the term "adolescents" and in other places "young adults." I would stick to one term for clarity and consistency. Also, the language of the themes is slightly different in the abstract/results/discussion. I find it helps the same language is used throughout.

Thank you very much for your constructive and encouraging review of our manuscript. We particularly appreciate your positive comments and helpful suggestions. We have taken these comments into account and have made appropriate changes to the manuscript.

First, we have clarified our terminology regarding the developmental stage considered in our manuscript in line with WHO definitions (i.e., adolescents and young people). These terms are now used consistently throughout the updated manuscript as appropriate (e.g., if studies only include adolescent samples, we use the word adolescent; since our results mainly focus on adolescent experiences, we use the term adolescent in the results section). However, throughout the rest of the manuscript we have adopted more neutral terminology where possible (e.g., changing “adolescent” or “young people” for “participant” or “individual”):

REVISED MANUSCRIPT (p. 3, lines 36-38): “According to the World Health Organisation, adolescence encompasses the period from 10-19 years old, while the term young people refers to individuals between 10-24 years old (WHO, 2001).”

Second, we have also ensured consistency in how the themes were discussed throughout the different sections of the manuscript. Particularly we made sure to introduce the same theme title in the abstract as it has been used in the results and discussion.

REVISED MANUSCRIPT (p.2 11-13): “Three themes were developed which consider 1) the role of friendships in self-harm progression; 2) the role of self-harm in friendship evolution; and 3) the meaning of friendships in the context of adolescents’ self-harm.”

6. PLOS authors have the option to publish the peer review history of their article ([what does this mean?](https://journals.plos.org/plosone/s/editorial-and-peer-review-process#loc-peer-review-history)). If published, this will include your full peer review and any attached files.


If you choose “no”, your identity will remain anonymous but your review may still be made public.


**Do you want your identity to be public for this peer review?** For information about this choice, including consent withdrawal, please see our [Privacy Policy](https://www.plos.org/privacy-policy).

Reviewer #1: No

Reviewer #2: No

[NOTE: If reviewer comments were submitted as an attachment file, they will be attached to this email and accessible via the submission site. Please log into your account, locate the manuscript record, and check for the action link "View Attachments". If this link does not appear, there are no attachment files.]

While revising your submission, please upload your figure files to the Preflight Analysis and Conversion Engine (PACE) digital diagnostic tool, <https://pacev2.apexcovantage.com/>. PACE helps ensure that figures meet PLOS requirements. To use PACE, you must first register as a user. Registration is free. Then, login and navigate to the UPLOAD tab, where you will find detailed instructions on how to use the tool. If you encounter any issues or have any questions when using PACE, please email PLOS at <a href="mailto:figures@plos.org">figures@plos.org. Please note that Supporting Information files do not need this step.
